# Effects of Cardiovascular Risk Factors on Cardiac STAT3

**DOI:** 10.3390/ijms19113572

**Published:** 2018-11-12

**Authors:** Márton Pipicz, Virág Demján, Márta Sárközy, Tamás Csont

**Affiliations:** Metabolic Diseases and Cell Signaling (MEDICS) Research Group, Department of Biochemistry, Interdisciplinary Excellence Centre, University of Szeged, Dóm tér. 9., H-6720 Szeged, Hungary; pipicz.marton@med.u-szeged.hu (M.P.); demjan.virag@med.u-szeged.hu (V.D.); sarkozy.marta@med.u-szeged.hu (M.S.)

**Keywords:** STAT family, comorbidity, myocardial infarction, coronary artery disease, cardioprotection, mPTP, JAK

## Abstract

Nuclear, mitochondrial and cytoplasmic signal transducer and activator of transcription 3 (STAT3) regulates many cellular processes, e.g., the transcription or opening of mitochondrial permeability transition pore, and its activity depends on the phosphorylation of Tyr705 and/or Ser727 sites. In the heterogeneous network of cardiac cells, STAT3 promotes cardiac muscle differentiation, vascular element formation and extracellular matrix homeostasis. Overwhelming evidence suggests that STAT3 is beneficial for the heart, plays a role in the prevention of age-related and postpartum heart failure, protects the heart against cardiotoxic doxorubicin or ischaemia/reperfusion injury, and is involved in many cardioprotective strategies (e.g., ischaemic preconditioning, perconditioning, postconditioning, remote or pharmacological conditioning). Ischaemic heart disease is still the leading cause of death worldwide, and many cardiovascular risk factors contribute to the development of the disease. This review focuses on the effects of various cardiovascular risk factors (diabetes, aging, obesity, smoking, alcohol, depression, gender, comedications) on cardiac STAT3 under non-ischaemic baseline conditions, and in settings of ischaemia/reperfusion injury with or without cardioprotective strategies.

## 1. Introduction

Signal transducer and activator of transcription 3 (STAT3) has a central role in transmitting extracellular signals from the plasma membrane to the nucleus and mitochondria, where it influences transcription and mitochondrial function, thereby regulating diverse biological processes.

### 1.1. Structure of STAT3

The protein encoded by mammalian STAT3 gene contains six functional domains: N-terminal, coiled-coil, DNA-binding, linker, SH2 and C-terminal transactivation domains [1]. There are two phosphorylation sites to activate STAT3: a tyrosine residue on the SH2 domain (Tyr705) and a serine residue on the transactivation domain (Ser727). As a result of alternative splicing two major isoforms exist: the abundant STAT3alpha (770 amino acids) and less abundant STAT3beta (722 amino acids) lacking C-terminal transactivation domain and Ser727 phosphorylation site [1]. 

### 1.2. Signalling

**Figure 1:**
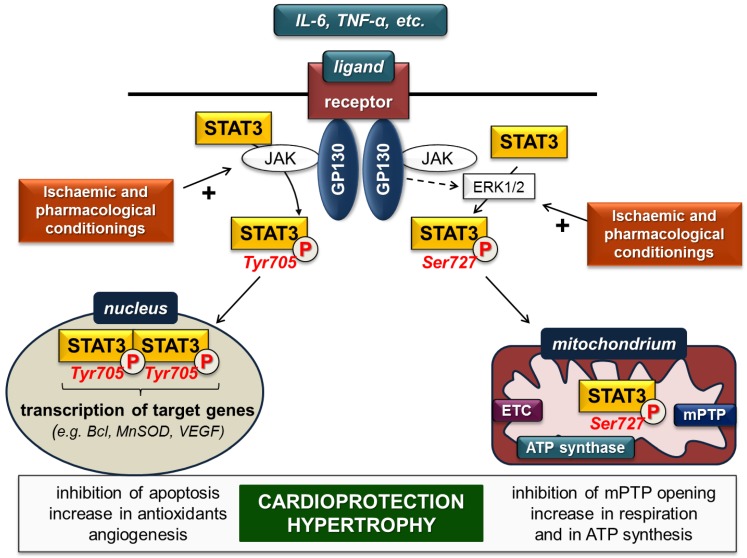
STAT3 signalling in cardiac myocytes in settings of ischaemia/reperfusion. ETC: electron transport chain; GP: glycoprotein; JAK: Janus kinase; mPTP: mitochondrial permeability transition pore; MnSOD: manganese-dependent superoxide dismutase; VEGF: vascular endothelial growth factor. (P in circle represents phosphorylated STAT3 forms; dashed arrow indicates several steps; plus (+) sign represents activation).

A wide variety of extracellular polypeptide ligands like interleukin-(IL)-6-family, leukaemia inhibitory factor, oncostatin M, ciliary neurotrophic factor, and cardiotrophin acts on plasma membrane receptors and activate STAT3, predominantly via the glycoprotein-130-Janus kinase (gp-130-JAK) axis [2]. Ligand binding by the receptor complex leads to activation of receptor-associated JAKs, thereby activating STAT3 directly and/or non-directly via the extracellular signal-regulated kinase 1 and 2 (ERK1/2) pathway (Figure 1).

Direct activation occurs once gp-130 is phosphorylated by JAK and provides binding sites for the SH2 domain of STAT3. After recruitment of STAT3, it is phosphorylated and activated by JAKs on Tyr705. Subsequently, STAT3 dissociates from the receptor complex, forms homo- or heterodimers with other STAT proteins, and translocates into the nucleus, where it binds to consensus DNA sequences (so-called gamma-interferon-activated sites (GAS)) and finally initiates transcription of target genes [2]. STAT3 enhances expression of several genes encoding, for instance, anti-apoptotic (e.g., Bcl-xl, MCL-1), anti-oxidant (e.g., MnSOD, metallothionein) and pro-angiogenic (e.g., VEGF) proteins.

The gp-130-JAK axis simultaneously activates ERK1/2, which in turn phosphorylates the Ser727 site of STAT3 monomers and/or Tyr705-phosphorylated STAT3 dimers to influence the dimer’s transcriptional activity. Phosphorylation of STAT3 monomers on Ser727 leads to translocation into the mitochondria without dimerization [3,4]. In the mitochondria, Ser727-STAT3 interacts with complex I of the electron transport chain, the mediator of mitochondrial permeability transition pore (mPTP) cyclophilin D, mitochondrial DNA and may have an impact on complex II and complex V (ATP synthase). Therefore, mitochondrial STAT3 influences ATP synthesis, the opening of mPTP, production of reactive oxygen species and mitochondrial transcription [3,4].

Besides classic ligands, STAT3 signalling is activated or modulated by other endogenous or exogenous peptides including insulin, leptin, angiotensin II, erythropoietin, granulocyte stimulating factor, etc.

### 1.3. STAT3 in the Heart

STAT3 is expressed in different cell types of the heart such as cardiomyocytes, cardiac fibroblasts, endothelial cells, smooth muscle cells, inflammatory cells and cardiac neurons. In the heterogeneous network of cardiac cells STAT3 regulates cell-to-cell communication (for review, see [5]). For instance, STAT3 promotes cardiac muscle differentiation [6] and vascular element formation in the heart [7], regulates β-adrenergic functions [8] and extracellular matrix homeostasis [9]. Overwhelming evidence suggests that STAT3 is beneficial for the heart [10]. STAT3 transduces hypertrophic signals under physiological (e.g., pregnancy) and pathophysiological (e.g., pressure overload) remodelling [11], plays a role in the prevention of age-related and postpartum heart failure, and protects the heart against cardiotoxic doxorubicin and ischaemic injury [2]. 

Ischaemic heart disease is still a major cause of death and disability worldwide; therefore, cardioprotective strategies and revealing of signalling are needed. Ischaemic conditioning is a manoeuvre for protecting the heart against the detrimental effects of ischaemia/reperfusion injury by means of the application of brief non-harmful ischaemia/reperfusion cycles to elicit endogenous cardioprotective mechanisms. When the conditioning method is applied before, under or after the prolonged lethal ischaemia, it is called ischaemic preconditioning, perconditioning or postconditioning, respectively. Research into the underlying molecular mechanisms of cardioprotection results in investigation of pharmacological agents that mimic the cardioprotective effect of ischaemic conditioning (pharmacological conditioning).

Several studies have reported that ischaemia enhances STAT3 phosphorylation [12,13,14], and activation of STAT3 protects the myocardium against ischaemia/reperfusion injury (Figure 1, for review, see [15]). Moreover, STAT3 is involved in many cardioprotective ischaemic and pharmacological conditioning methods, for instance in ischaemic preconditioning [16], perconditioning [17] or postconditioning [18] (for review, see [19]).

Many cardiovascular risk factors contribute to the development of ischaemic heart disease and interfere with the cardioprotective effect of conditioning manoeuvres (for review, see [20]). Therefore, it is feasible to speculate that risk factors may unfavourably alter protective signalling mechanisms in the heart (e.g., reperfusion injury salvage kinase signalling or survivor activating factor enhancement pathways), thereby resulting in unwanted cardiac consequences. This review focuses on the effects of various cardiovascular risk factors (diabetes, aging, obesity, smoking, alcohol, depression, gender, comedications) on cardiac STAT3, a well-known cardioprotective signal molecule, under non-ischaemic baseline conditions and in settings of ischaemia/reperfusion injury with or without cardioprotective strategies. We systemically analysed phosphorylation (p-STAT3, at Tyr705 and Ser727 sites) and expression (total-STAT3) of cardiac STAT3. Furthermore, the ratio of p-STAT3 and total-STAT3 was considered as STAT3 activation.

## 2. Effect of Cardiovascular Risk Factors on Cardiac STAT3 under Non-Ischaemic Baseline Conditions

### 2.1. Diabetes

Diabetes mellitus is a condition characterized by an increased blood glucose level. It is one of the most prevalent metabolic diseases worldwide and is also associated with increased mortality. In 2017, an estimated 425 million people suffered from diabetes, a number that is expected to increase by 48% within three decades according to the International Diabetes Federation [21]. Diabetic patients have a greater chance of developing cardiovascular complications including ischaemic heart disease such as the life-threatening acute myocardial infarction [22], since diabetes exerts harmful effects on the cardiovascular system, resulting in cardiomyopathy [23] and vasculopathy [24]. An explanation for this may be that diabetes leads to molecular changes in the heart, e.g., marked alteration of cardiac gene expression profile in various experimental models [25,26,27].

Based on a literature review, the effect of diabetes on non-ischaemic baseline expression, phosphorylation or activation of cardiac STAT3 protein seems to be rather controversial. Several publications demonstrated a significant decrease in cardiac STAT3 phosphorylation and/or activation in various experimental models of diabetes (Table 1). The first study to demonstrate this phenomenon was written by Wang and colleagues [28]. They induced type I diabetes in male Sprague-Dawley rats by streptozotocin (STZ) injection. After five weeks of diabetes induction, they found that myocardial STAT3 activation (at the Ser727 site) was lower in the diabetic group. Later the same research group confirmed these results in STZ-induced type I diabetes models [29,30] and demonstrated that phosphorylation and activation of STAT3 at Tyr705 was also decreased in diabetes [29,30,31]. Besides type I diabetes models, reduction of cardiac STAT3 expression was also described in a type II diabetes model, i.e., STAT3 mRNA was downregulated in non-obese Goto-Kakizaki rats [25]. Moreover, similar trends were observed in in vitro models of diabetes. In H9c2 cells subjected to high glucose conditions (25 mM glucose added to the medium), the non-ischaemic baseline STAT3 phosphorylation (Tyr705 and/or Ser727) and activation was significantly decreased [29,32]. Similarly, exposure of isolated adult rat ventricular cardiomyocytes to high glucose conditions also resulted in reduced non-ischaemic baseline phosphorylation and activation of STAT3 (Tyr705) [33]. 

In contrast to the results above, a few studies found that the non-ischaemic baseline level of phosphorylated STAT3 significantly increased in diabetic hearts (Table 1). For instance, the activation of cardiac STAT3 was increased four weeks after the induction of diabetes with STZ injection in C57BL/6 mice [34]. In a longer, 21-week study, a similar increase in phosphorylated STAT3 was observed in Wistar rats, which were first fed a high-glucose and high-fat diet, then injected with STZ [35]. Furthermore, in a Sprague-Dawley rat model of STZ-induced diabetes, a significant increase in non-ischaemic baseline STAT3 activation has been reported [36]. In addition, increases in STAT3 expression was also demonstrated in some studies [36,37]. Baseline phosphorylation and/or activation STAT3 level also increased in some in vitro studies, such as in H9c2 cells [36] or in primary rat cardiac fibroblasts [38] subjected to a high glucose condition. Additionally, increased non-ischaemic baseline STAT3 expression was observed in H9c2 cells [37].

Recently some potential cardioprotective agents have been suggested to attenuate STAT3 dysregulation in diabetes (Table 2). The impaired activation or phosphorylation of STAT3 due to STZ-induced diabetes was shown to be restored by N-acetylcysteine [28,30]. In a rat model of diabetes induced by a high-glucose and -fat diet + STZ injection, enhanced phosphorylation of STAT3 due to diabetes was attenuated by losartan treatment [35]. In another study using an STZ-induced rat diabetes model, telmisartan attenuated STAT3 expression, which was increased by diabetes [37]. 

In summary, the findings regarding the effect of diabetes on cardiac STAT3 phosphorylation, expression and activation are inconsistent. The reasons for these controversies are unclear and may include substantial differences in the method of induction, type, severity, and duration of diabetes as well as differences in the method of detection of STAT3 phosphorylation and expression.

### 2.2. Obesity

The definition of obesity among adults is based on a standard cutoff point of body mass index (i.e., BMI ≥ 30 kg/m^2^) [39]. Obesity is one of the top health concerns worldwide. According to a global survey in 195 countries, 604 million adults and 108 million children were obese [39]. Obesity is a well-known risk factor for cardiovascular diseases [39,40,41]. In obesity, the adipose tissue is unable to store more extra fat, which results in lipid overflow to other organs, such as the liver, pancreas skeletal muscle and heart [42]. Obese individuals are typically predisposed to increased heart rate and stroke volume, progress to ischaemic cardiomyopathy, compensatory left ventricular hypertrophy and remodelling, and later dilated cardiomyopathy with cardiac fibrosis and apoptosis [43]. Obesity is often accompanied by dyslipidemias, hypertension, and insulin resistance, leading to metabolic syndrome. 

The adipose tissue produces various adipokines such as leptin, which is involved in the regulation of appetite. Obesity is associated with elevated circulating leptin levels and hypothalamic leptin resistance [44]. Clinical studies demonstrated a positive correlation between serum leptin levels and left ventricular hypertrophy independent of blood pressure values [45,46]. Leptin and its receptor are expressed in the heart, and leptin has been shown to promote left ventricular hypertrophy [47,48,49]. The leptin receptor belongs to cytokine type I receptors, which are known to signal via activation of the JAK2/STAT3 pathway [50]. The activation of the leptin-STAT3 signalling by high-fat diet was reported to be associated with hypertrophy and increased expression and activation of cardiac STAT3 in C57BL/6 mice, while STAT3 activation remained unchanged in leptin-receptor-deficient db/db mice [51,52] (Table 3). Another study demonstrated increased STAT3 phosphorylation and expression in the hearts of Zucker rats, a genetic model of obesity [53]. Moreover, increased STAT3 expression has been shown to contribute to the development of left ventricular hypertrophy in hypercholesterolemic hamsters [54] and pigs suffering from metabolic syndrome [55]. In contrast, another study demonstrated that metabolic syndrome mimicked by high glucose, salt, and cholesterol treatment in cardiomyocyte-like H9c2 cells reduced viability and STAT3 activation [56]. In Sprague-Dawley rats, a high-fat diet resulted in decreased phosphorylation and expression of cardiac STAT3, along with an increased sensitivity to doxorubicin-induced cardiotoxicity [57]. Two additional studies showed no alteration of cardiac STAT3 activation and phosphorylation and expression in high-fat-diet-induced obese rats [51] or leptin-receptor-deficient obese mice (ob/ob) [58], respectively.

A preclinical study proved that cardiac ciliary neurotrophic factor (CNTF) ameliorated left ventricular hypertrophy in leptin-deficient ob/ob and leptin-resistant db/db mice via the STAT3 and ERK1/2 signalling pathway by activation of CNTF receptor that is structurally similar to the leptin receptor [59]. 

Altogether, the majority (but not all) of studies showed no change or increase in cardiac STAT3 phosphorylation, expression and/or activation in animal models of experimental obesity and/or hyperlipidemia. Based on the literature data, it seems that facilitated cardiac STAT3 signalling might contribute to activation of hypertrophic and surviving pathways in obesity. 

### 2.3. Hypertension

According to the 2017 High Blood Pressure Clinical Practice Guideline, systolic blood pressure ≥130 and/or diastolic blood pressure ≥80 mmHg is considered as hypertension [60]. Observational studies have demonstrated graded associations between hypertension and increased cardiovascular risk to, e.g., myocardial infarction, heart failure, stroke, peripheral artery disease, etc. [61,62]. Moreover, patients with hypertension often have other cardiovascular risk factors such as hypercholesterolemia, obesity, diabetes mellitus, chronic kidney disease and smoking [63].

Left ventricular hypertrophy is a secondary consequence of hypertension and independently predicts future cardiovascular events [60,64]. Therefore, it is very difficult to separate the effects of hypertension, left ventricular hypertrophy and later systolic heart failure on cardiac STAT3 in preclinical and clinical studies. Nevertheless, cardiac phosphorylation of STAT3 has been reported to increase shortly after (peaking at 60 min) induction of pressure overload by constriction of the abdominal aorta [65] (Table 4). Among the signalling pathways that mediate cardiac hypertrophy and heart failure, the activation of the JAK/STAT pathway is thought to play a pivotal role in the response to various stimuli such as pressure overload, cytokines, neurohormones, growth factors, ischaemia, etc. [11]. Detailed mechanisms by which STAT3 interacts with a broad range of cellular and molecular mechanisms to induce left ventricular hypertrophy and heart failure have been discussed in recent review articles, so here we just refer to these excellent reviews [11,15,66,67,68].

### 2.4. Chronic Kidney Disease

Chronic kidney disease (CKD) is a clinical syndrome defined as persistent deterioration of kidney function [41]. The prevalence of all stages of CKD varies between 7% and 12% worldwide [69]. In CKD patients, cardiovascular diseases are the leading cause of death [70]. The high incidence of cardiovascular diseases in CKD can be attributed to different systemic complications of CKD [41]; for instance, hypocalcemia and hyperkalemia often lead to life-threatening arrhythmias [70]. Increased oxidative stress, systemic inflammation, accelerated atherosclerotic process and deteriorating arterial hypertension in CKD often results in cardiac hypertrophy, later progressing to heart failure [70]. 

Cardiac STAT3 phosphorylation increased in a rat model of doxorubicin-induced CKD and cardiac hypertrophy [71] (Table 4). This study also reported that 60 min daily of swimming or running for 11 weeks could attenuate cardiac hypertrophy through the cardiotrophin-1-LIFR-gp130-JAK/STAT3 pathway [71]. Swimming reduced cardiac STAT3 phosphorylation both in the control and CKD group, which might lead to the attenuation of cardiac hypertrophy in CKD animals [71]. 

Although alteration of cardiac STAT3 has been reported in CKD, whether this is due to direct effects or due to secondary effects via cardiovascular complications of CKD (e.g., cardiac hypertrophy and fibrosis) is very difficult to distinguish.

### 2.5. Aging

The aging human population is an epidemiological burden. It is estimated that more than 2 billion individuals will be over the age of 60 by 2050 worldwide [75]. The prevalence of cardiovascular diseases increases with age [76,77], and age might be a dominant risk factor in the elderly since the impact of many traditional risks (e.g., obesity or hypertension) decline with age [78]. 

The first evidence demonstrating a crucial role for cardiac STAT3 in aging was reported by Jacoby et al., showing the development of cardiac dysfunction and fibrosis with advancing age in mice with a cardiomyocyte-restricted deletion of STAT3 [79]. Later, reductions in STAT3 phosphorylation and expression in the right ventricle were shown in 13-month-old mice compared to three-month-old mice [18] (Table 5). Two years later, the same research group demonstrated that the expression of STAT3 in subsarcolemmal mitochondria is reduced in the left ventricles of 21-month-old mice [80]. In accordance with these findings, STAT3 activation was reduced in 12-month-old and 24-month-old rats versus six-month-old controls [81].

In contrast, some studies described no alteration or increase of cardiac STAT3 in association with advanced age. Phosphorylation was unchanged in 14-month-old mice [82] or 20‒24-month-old rats [83], and p-STAT3beta was not altered in 24-month-old mice [84] (Table 5). Three studies showed that the level of STAT3 expression was increased in response to aging in old mice [82], hamsters [72] and rats [73]; however, these findings are limited since the activation (i.e., phosphorylation) was not examined.

Although age-related STAT3 dysregulation in human hearts has not been reported, research on blood samples from two independent cohort studies showed that STAT3 was positively associated with age [85]. 

Regarding possible modulation of STAT3 dysregulation in aging, Castello et al. have reported that alternate-day fasting restored the decline in STAT3 activation in elderly rats to young values and protected the heart against age-related hypertrophy [81]. 

Taken together, it is controversial whether STAT3 expression or activation is affected by aging or not, and further research is needed to elucidate the phenomenon. Nevertheless, fasting seems to be effective for the restoration of aging-associated STAT3 dysregulation.

### 2.6. Smoking

Smoking has well-known detrimental effects on health. Both active and passive (secondhand) smoking are predominant risk factors for coronary heart disease [86]. We et al. reported that passive smoking increases cardiac STAT3 expression in young rats [73], but does not alter STAT3 expression in hamsters [72] (Table 4). In aged rats and hamsters exposed to passive smoking, cardiac STAT3 expression showed a tendency to increase [72,73]. 

### 2.7. Alcohol

Alcohol consumption is a widespread social habit. Although several studies showed potential benefits of moderate alcohol consumption on coronary heart diseases [87], heavy alcohol drinking may lead to the development of cardiomyopathy. In mice, chronic 4% alcohol liquid diet for 12 weeks induced cardiomyopathy and was associated with decreased STAT3 phosphorylation, which was reconciled in mice overexpressing aldehyde dehydrogenase 2 [74] (Table 4). This finding indicates a potential effect of alcohol on STAT3 signalling.

### 2.8. Comedications

Patients at risk of cardiovascular disease are often treated with various medications that may also contribute to alteration of cardiac molecular signalling. Administration of the lipid-lowering drug simvastatin (10 mg/kg single daily dose) for five days in male Wistar rats increased cardiac p-STAT3 without affecting t-STAT3 [88]. Controversially, simvastatin gavage (10 mg/kg single daily dose) for 30 days and then for seven days intraperitoneally did not affect p-STAT3 [89]. 

### 2.9. Summary

**Figure 2:**
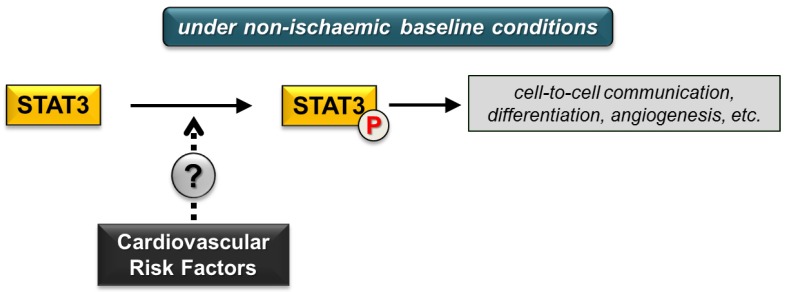
Cardiovascular risk factors and cardiac STAT3 activation under non-ischaemic baseline conditions. (P in circle represents phosphorylated STAT3 form; dotted arrow and question mark indicate inconclusive effects of risk factors).

The effect of various cardiovascular risk factors on cardiac STAT3 signalling under non-ischaemic baseline conditions remains inconclusive (Figure 2) due to limited literature data or conflicting findings. Future experimental studies focusing on this area may help us to draw adequate conclusions.

## 3. Effect of Cardiovascular Risk Factors on Cardiac STAT3 Activation in Settings of Ischaemia/Reperfusion

### 3.1. Diabetes

Diabetes is a well-known risk factor for the development of ischaemic heart disease. Moreover, clinical studies showed that diabetes mellitus increased the susceptibility of the myocardium to ischaemia/reperfusion injury and that the long-term outcome of ischaemic heart disease is worsened by diabetes. However, the effect of diabetes on the susceptibility of the myocardium to acute ischaemia/reperfusion injury is controversial in animal models. In experimental models of both type I or type II diabetes, infarct size was demonstrated to be significantly larger, unchanged, or significantly smaller in diabetic rats compared to nondiabetic controls [29,90,91,92]. 

In contrast to discrepancies regarding the effect of diabetes on infarct size, post-ischaemic phosphorylation/activation of cardiac STAT3 was clearly downregulated in experimental models of diabetes in all investigations (Table 6). In an animal model of STZ-induced type I diabetes, after 30 min ischaemia and 2 h reperfusion there was a significant reduction in phosphorylated STAT3 (Tyr705) levels in the diabetic group compared to the nondiabetic control group (tissue samples were collected from the ischaemic zone of the myocardium) [93]. This reduction of post-ischaemic STAT3 phosphorylation and/or activation due to diabetes was confirmed by several studies of another research group in the ischaemic tissue as well as in whole heart or ventricular tissue samples [29,31,33,92,94]. In addition, the phosphorylated STAT3 (Tyr705) and total STAT3 levels were significantly reduced in a type II diabetes model, i.e., in isolated perfused hearts of leptin receptor null, homozygous db/db mice subjected to ischaemia/reperfusion compared to wild-type hearts subjected to ischaemia/reperfusion [95].

The same trend was observed in in vitro models of diabetes or acute hyperglycaemia. In H9c2 cells subjected to high glucose conditions (25 mM glucose), the post-ischaemic STAT3 phosphorylation and activation (at Ser727 and Tyr705 sites) were significantly lower [29,32]. Similarly, exposure of isolated adult rat ventricular cardiomyocytes to high glucose conditions also resulted in reduced post-ischaemic STAT3 activation (at Tyr705 site) [33,92]. 

In summary, post-ischaemic STAT3 phosphorylation and/or activation are significantly decreased due to diabetes in all studies irrespective of the applied models, which may contribute to increased susceptibility to myocardial ischaemia/reperfusion injury in diabetes.

### 3.2. Obesity

Obese people are more prone to developing coronary artery disease [40]. Leptin signalling has been shown to ameliorate cardiac dysfunction and remodelling four weeks after myocardial infarction by increasing STAT3 phosphorylation in calorie-restricted lean and obese ob/ob mice [58] as well as in tamoxifen-inducible leptin receptor knockout mice [96] (Table 7). The obesity-associated hormone leptin has been shown to exert an infarct size-limiting effect after 35 min global ischaemia and 35 min reperfusion in non-obese C57Bl/6 mice [97]; however, leptin treatment was associated with reduced levels of phosphorylated and total STAT3 after myocardial infarction in this study [97]. Another study demonstrated that cardiac STAT3 activation was not altered due to diet-induced hypercholesterolemia in rabbit hearts subjected to 30 min ischaemia and 10 min reperfusion [98]. 

### 3.3. Chronic Kidney Disease

The hypertrophic and fibrotic myocardium is more sensitive to ischaemia; therefore, acute myocardial infarction is a common cause of cardiovascular morbidity and mortality in CKD patients [70]. To date, only one study has reported data on cardiac STAT3 in experimental CKD. Neither expression nor activation of cardiac STAT3 was affected by CKD in a rat model of in vivo ischaemia/reperfusion [99] (Table 7).

### 3.4. Aging

Aging aggravates myocardial ischaemia/reperfusion injury in humans [100] and rodents [100,101,102,103]. An experimental study showed reduced STAT3 activation in the left ventricle at reperfusion after regional ischaemia in 13-month-old mice compared to three-month-old mice [18] (Table 7). Another study found unaltered activation of cardiac STAT3 in aged rats subjected to 30 min ischaemia and 15 min reperfusion compared to young controls undergoing ischaemia/reperfusion [83].

### 3.5. Gender

It is well known that the risk of cardiovascular diseases is higher in males compared to females. The molecular and cellular basis of the cardiovascular gender difference has been reviewed elsewhere [104]. Scientific evidence suggests that the female sex hormone oestrogen exerts a cardioprotective effect, which also explains why postmenopausal women have a higher cardiovascular risk compared to younger females [105]. 

Wang et al. demonstrated that in hearts isolated from male wild-type C57BL/6J mice and subjected to ex vivo global ischaemia/reperfusion, the STAT activation was lower compared to hearts from female mice [106] (Table 7). This difference was also associated with better functional recovery after ischaemia/reperfusion in female mice [106]. Myocardial STAT3 activation after ex vivo ischaemia/reperfusion was also attenuated in the hearts of male Sprague-Dawley rats compared to females [107]. However, in the same study, in hearts of castrated male rats subjected to ischaemia/reperfusion, the myocardial STAT3 activation was higher compared to hearts of male controls, possibly due to lower levels of endogenous testosterone. Moreover, exogenous testosterone administration decreased activation of STAT3 in hearts of castrated males as well as in females compared to males; therefore, it was concluded that testosterone has a negative effect on myocardial STAT3 activation after ischaemia/reperfusion [107].

### 3.6. Depression

Depression is common in patients with coronary heart disease and is associated with increased cardiovascular mortality [108]. Depression was not shown to influence STAT3 activation in an ex vivo model of regional ischaemia/reperfusion in hearts isolated from Sprague-Dawley rats exposed to experimental depression induced by chronic mild stress [109] (Table 7).

### 3.7. Comedications

Administration of the lipid-lowering drug simvastatin (10 mg/kg single daily dose) for five days in male Wistar rats did not affect ischaemia/reperfusion-induced STAT3 phosphorylation [88]. 

## 4. Effect of Cardioprotective Strategies against Ischaemia/Reperfusion on Cardiac STAT3 Activation in the Presence of Cardiovascular Risk Factors

### 4.1. Diabetes

In the literature, studies exist reporting both preserved as well as impaired cardioprotection by ischaemic or pharmacological conditionings [91]. Nevertheless, ischaemic pre- and postconditioning mechanisms, which aim to attenuate ischaemia/reperfusion injury, were inefficient or required extra ischaemia/reperfusion cycles to induce cardioprotection in the majority of animal models of chronic diabetes [90,110,111]. The efficacy of pharmacological preconditioning was also impaired in diabetes, for instance in the case of isoflurane [112] or l-glutamate [113]. In contrast, the infarct-size-limiting effect of remote preconditioning induced by repeated non-invasive limb ischaemia was preserved in STZ-induced diabetes [29].

Several studies have demonstrated that the presence of diabetes attenuated phosphorylation and/or activation of STAT3 in hearts undergoing ischaemia/reperfusion with ischaemic [31], pharmacological [29,33,93,94], or remote [29] conditioning when compared to nondiabetic controls subjected to ischaemia/reperfusion with corresponding conditioning (Table 8). In these studies various ischaemic or pharmacological conditioning either increased or did not change STAT3 activation in the diabetic state. These studies suggest that diabetes aggravates the stimulatory effect of various conditioning on the phosphorylation/activation of cardiac STAT3. Similar pattern was shown in an in vitro model of hyperglycaemia in H9c2 cells [29]. 

Recently, some promising agents have been suggested to prevent STAT3 dysregulation in diabetes. In diabetic mice, rapamycin [95] (Table 8) and its nanoformulated form, Rapatar [114] (Table 9), increased the phosphorylation and activation of STAT3 (Tyr705), which could contribute to the infarct-size-reducing effect of these treatments. The combination of antioxidant N-acetylcysteine and allopurinol restored the decreased levels of p-STAT3 (Ser727, Tyr705) after ischaemia/reperfusion and so contributed to smaller infarct size in STZ-induced diabetes [92]. N-acetylcysteine treatment together with sevoflurane postconditioning has the same beneficial effects [94] (Table 9). In some studies, the phosphorylation/activation of STAT3 was also increased in response to various cardioprotective agents in diabetes models [32,92,94,114] (Table 9).

In summary, the majority of articles revealed that diabetes attenuates ischaemic or pharmacological conditioning-induced cardiac STAT3 activation after ischaemia/reperfusion. This could be related to the loss of cardioprotection and increased infarct size in diabetes. Interestingly, there are some potential therapeutic agents like the antioxidant N-acetylcysteine and allopurinol or rapamycin that can restore the STAT3 phosphorylation and so can contribute to attenuated myocardial damage.

### 4.2. Obesity

There is a shortage of studies investigating the effect of obesity on ischaemic or pharmacological conditioning-induced cardiac STAT3 activation after ischaemia/reperfusion. Nevertheless, an experimental study demonstrated that cardiac STAT3 activation was increased in rabbit hearts subjected to two cycles of preconditioning with 5 min ischaemia/10 min reperfusion followed by 30 min ischaemia and 10 min reperfusion as compared to the ischaemia/reperfusion control group [98]. In this study, the natural olive constituent oleuropein induced nutritional cardioprotection in normal and cholesterol-fed rabbits by activating the STAT3 signalling pathway, which was similar to the effects seen in ischaemic preconditioning hearts [98] (Table 10).

### 4.3. Chronic Kidney Disease

There are only limited data on the possible interaction of CKD with cardioprotective strategies. Byrne et al. reported that ischaemic conditioning still reduces infarct size after four weeks of subtotal nephrectomy in male Wistar rats [99]. This study found that (i) the level of phosphorylated STAT3 was significantly increased by ischaemic preconditioning both in the CKD and controls, (ii) the level of STAT3 expression was not different in response to CKD or ischaemic preconditioning, and (iii) the activation of STAT3 was significantly increased due to ischaemic preconditioning in both the CKD and control animals [99] (Table 10). Since the relevance of these findings were challenged due to the short duration of kidney disease [115], our research group used male Wistar rats after 29 weeks of subtotal nephrectomy and demonstrated that ischaemic preconditioning was still cardioprotective in a chronic uremic condition [116]. Nevertheless, cardiac STAT3 has not been investigated in this model [116].

### 4.4. Aging

Several clinical [117,118] and animal [18,119] studies showed that the beneficial effect of cardioprotective approaches is lost in aged hearts (for review, see [120,121]). To date, one study was conducted on revealing the association of STAT3 dysregulation and loss of cardioprotection by ischaemic conditioning. In the aged mouse heart, ischaemic postconditioning was ineffective and associated with reduced cardiac STAT3 activation. Therefore, the authors concluded that reduced STAT3 activation might contribute to the age-related loss of protection [18] (Table 10).

### 4.5. Depression

Depression is common in patients with coronary heart disease and is associated with increased cardiovascular mortality [108]. Endothelial protection induced by ischaemic postconditioning disappears in patients with major depression [122]. Furthermore, ischaemic postconditioning was ineffective in rats with chronic depression induced by three-week mild stress [109]. In hearts of non-depressed animals, ischaemic postconditioning enhanced STAT3 activation; however, postconditioning failed to increase STAT3 activation in depressed condition (Table 10). The authors concluded that impaired activation of STAT3 may contribute to a loss of cardioprotection [109]. 

### 4.6. Comedications

Certain pharmacological agents used for preventing ischaemic heart disease (e.g., glyceryl trinitrate, statins) have been reported to interfere with the cardioprotective effect of conditionings [123,124,125]. Rosuvastatin administered on the day before in vivo myocardial infarction (8 mg/kg single dose), and thereafter at 4 mg/kg orally for five weeks, enhanced p-STAT3/t-STAT3 in the peri-infarct area [126].

## 5. Conclusions and Future Perspectives

Here, we reviewed current knowledge regarding the effect of various cardiovascular risk factors on STAT3 in the heart under non-ischaemic baseline conditions, and in settings of ischaemia/reperfusion injury with or without cardioprotective strategies (i.e., ischaemic pre- or postconditioning, as well as remote or pharmacological conditioning). Our conclusions may be somewhat limited as, in the case of most risk factors, only a few studies have focused primarily on the alteration of cardiac STAT3 due to a specific risk factor (e.g., hypertension, chronic kidney disease, smoking, alcohol consumption). Therefore, it is difficult to find strong evidence in those areas, and further studies are urged to investigate the effect of these risk factors on myocardial STAT3 signalling.

Nevertheless, in this review we highlighted that under non-ischaemic baseline conditions the STAT3 phosphorylation in response to risk factors is inconsistent (e.g., diabetes, obesity, aging) (Figure 2). The reason for this is unclear and may include differences in the species and strain of animals, the type, severity, and duration of risk factor condition as well as differences in the method of detection of STAT3 phosphorylation and expression.

**Figure 3:**
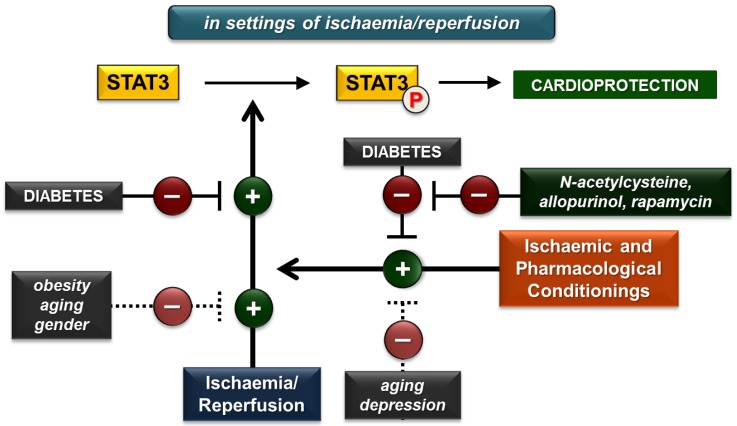
Summary of the effects of various cardiovascular risk factors on cardiac STAT3 activation due to ischaemia/reperfusion and ischaemic or pharmacological conditionings. (P in circle represents phosphorylated STAT3 form. Dotted lines indicate solely proposed effects due to insufficient number of studies. Plus (+) and minus (−) signs represent activation and inhibition, respectively).

More interestingly, most of the findings indicate that certain risk factors (e.g., diabetes, obesity, aging, male gender) attenuate the activation of cardiac STAT3 in settings of ischaemia/reperfusion, which may contribute to a worsening of the ischaemic tolerance of the heart. Moreover, based on our review of the literature, it seems that risk factors including diabetes, aging and depression decrease, which likely plays a role in the loss of cardioprotection (Figure 3). These results point out the therapeutic potential of restoring STAT3 dysregulation. Indeed, there are some potential therapeutic agents like the antioxidant N-acetylcysteine and allopurinol or rapamycin that beneficially affect STAT3 dysregulation in diabetes. However, the availability of compounds directly and selectively targeting STAT3 phosphorylation is currently very limited, and development of such agents would facilitate further research on the feasibility of STAT3 modulation as a cardioprotective intervention. Moreover, pharmacological STAT3 activation in relation to ischaemia/reperfusion (especially in the presence of risk factors) should be cardioselective and temporary due to the fact that prolonged upregulation of STAT3 in non-cardiac tissues is associated with various malignancies.

In summary, there is still no consensus in this research field and further focused studies are needed to elucidate the role of cardiovascular risk factors in dysregulation of myocardial STAT3 under different physiological and pathophysiological conditions. Further testing of the therapeutic potential of STAT3 activation in cardiac ischaemia/reperfusion in the presence of various cardiovascular risk factors should also be straightforward.

## Figures and Tables

**Table 1:** Effect of diabetes on cardiac STAT3 under non-ischaemic baseline conditions.

Animal or Cell	Test Group	Control Group	Tissue Sample	p-STAT3/t-STAT3 Activation	p-STAT3 Phosphorylation		t-STAT3 Expression	Conclusions	Ref.
Rat Sprague Dawley Male	STZ-induced diabetes	nondiabetic	ventricles	↓	↓ (NC)	Ser727	– (NC)	diabetes decreases STAT3 activation	[28]
			LV	↓	↓	Tyr705 Ser727	– (NC)	diabetes decreases STAT3 activation and phosphorylation	[29]
			whole heart	↓ (NC)	↓	Tyr705 Ser727	–	diabetes decreases STAT3 phosphorylation	[30]
				↓	↓	Tyr705	– (NC)	diabetes decreases STAT3 activation and phosphorylation	[31]
				↑	↑	?	↑	diabetes increases STAT3 activation, phosphorylation and expression	[36]
				N.D.	N.D.	N.A.	↑	diabetes increases STAT3 expression	[37]
Rat prague Dawley Male	isolated adult diabetic rat cardiomyocytes	isolated adult nondiabetic rat cardiomyocytes	cells	↓	↓	Tyr705	– (NC)	diabetes decreases STAT3 activation and phosphorylation	[33]
H9c2 cells	high glucose conditions	normal glucose condition	cells	↓	↓	Tyr705	– (NC)	high glucose condition decreases STAT3 activation and phosphorylation	[32]
				↓	↓	Tyr705 Ser727	– (NC)	high glucose condition decreases STAT3 activation and phosphorylation	[29]
				↑	↑ (NC)	?	↑ (NC)	high glucose condition increases STAT3 activation	[36]
				N.D.	N.D.	N.A.	↑	diabetes increases STAT3 expression	[37]
Rat Wistar N.A.	isolated cardiac fibroblasts in high glucose conditions	normal glucose condition	neonatal cells	↑	↑	?	– (NC)	high glucose condition increases STAT3 activation and phosphorylation	[38]
Rat Wistar Male	high-glucose and -fat diet + STZ-induced diabetes	nondiabetic	whole heart	↑ (NC)	↑	?	– (NC)	diabetes increases STAT3 phosphorylation	[35]
Mouse *C57BL/6* Male	STZ-induced diabetes	nondiabetic		↑	↑ (NC)	?	– (NC)	diabetes increases STAT3 activation	[34]

↓ in green cells: decrease; ↑ in red cells: increase; – in blue cells: no change; NC: not confirmed; N.D.: not detected; N.A.: not applicable; ?: phosphorylation site was not specified; LV: left ventricle; STZ: streptozotocin; p-STAT3: phosphorylated STAT3; t-STAT3: total STAT3.

**Table 2:** Effect of pharmacological treatments on non-ischaemic baseline cardiac STAT3 in diabetes.

Animal or Cell	Test Group	Control Group	Tissue Sample	p-STAT3/t-STAT3 Activation	P-STAT3 Phosphorylation		t-STAT3 Expression	Conclusions	Ref.
Rat *Sprague Dawley* Male	STZ-induced diabetes + N-acetylcysteine	STZ-induced diabetes	ventricles	↑	↑ (NC)	Ser727	– (NC)	N-acetylcysteine restores impaired activation of STAT3 in diabetes	[28]
			whole heart	↑ (NC)	↑	Tyr705 Ser727	–	N-acetylcysteine restores impaired phosphorylation of STAT3 in diabetes	[30]
Rat *Wistar* Male	high-glucose and -fat diet + STZ-induced diabetes + losartan	high-glucose and -fat diet + STZ-induced diabetes	whole heart	↓ (NC)	↓	?	– (NC)	losartan attenuates enhanced phosphorylation of STAT3 in diabetes	[35]
Rat *Sprague Dawley* Male	STZ-induced diabetes + telmisartan	STZ-induced diabetes	whole heart	N.D.	N.D.	N.A.	↓	telmisartan attenuates enhanced expression of STAT3 in diabetes	[37]

↓ in green cells: decrease; ↑ in red cells: increase; – in blue cells: no change; NC: not confirmed; N.D.: not detected; N.A.: not applicable; ?: phosphorylation site was not specified; LV: left ventricle; STZ: streptozotocin; p-STAT3: phosphorylated STAT3; t-STAT3: total STAT3.

**Table 3:** Effect of obesity on cardiac STAT3 under non-ischaemic baseline conditions.

Animal or Cell	Test Group	Control Group	Tissue Sample	p-STAT3/t-STAT3 Activation	p-STAT3 Phosphorylation		t-STAT3 Expression	Conclusions	Ref.
Mouse *C57BL/6* Unknown gender	high-fat-diet-induced obese mice	non-obese	ventricles	↑	↑ (NC)	Tyr705	– (NC)	high-fat diet increases STAT3 activation	[52]
	leptin-receptor-deficient (db/db) obese mice	non-obese	ventricles	–	– (NC)	Tyr705	– (NC)	STAT3 is not activated in db/db obesity	
Rat Sprague-Dawley Male	high-fat-diet-induced obese rats	non-obese	whole heart	–	– (NC)	?	– (NC)	high-fat diet did not influence STAT3 activation	[51]
Rat Zucker Male	leptin receptor deficient (fa/fa) obese rats	non-obese	LV	↑ (NC)	↑	?	↑	fa/fa genetic obesity increases STAT3 phosphorylation and expression	[53]
Hamster Golden Syrian Male	0.2% cholesterol diet-induced hypercholesterolemic hamsters	normo-cholesterolemia	LV	N.D.	N.D.	N.A.	↑	hypercholesterolemia increases STAT3 expression	[54]
Pig Bama miniature Female/Male	high-fat and high-sucrose diet-induced metabolic syndrome	non-metabolic syndrome	LV	N.D.	N.D.	N.A.	↑ *	metabolic syndrome increases STAT3 mRNA expression	[55]
H9c2 cells	metabolic syndrome induced by high glucose, salt, and cholesterol treatment	normal medium	cells	**↓** (ELISA)	N.E.	?	N.E.	metabolic syndrome decreases STAT3 activation	[56]
Rat Sprague-Dawley Male	high-fat-diet-induced obese rats	non-obese	whole heart	↓ (NC)	↓	?	↓	high-fat diet decreases STAT3 phosphorylation and expression	[57]
Mouse Leptin-receptor-deficient (ob/ob) obese Male	leptin-receptor-deficient (ob/ob) obese mice	non-obese	whole heart	– (NC)	–	Tyr705	–	STAT3 is not activated in ob/ob obesity	[58]

↓ in green cells: decrease; ↑ in red cells: increase; – in blue cells: no change; NC: not confirmed; N.D.: not detected; N.A.: not applicable; ?: phosphorylation site was not specified; LV: left ventricle; STZ: streptozotocin; p-STAT3: phosphorylated STAT3; t-STAT3: total STAT3; *: mRNA expression; ELISA: enzyme-linked immunosorbent assay.

**Table 4:** Effect of hypertension, chronic kidney disease, smoking and alcohol on cardiac STAT3 under non-ischaemic baseline conditions.

Animal or Cell	Test Group	Control Group	Tissue Sample	p-STAT3/t-STAT3 Activation	p-STAT3 Phosphorylation		t-STAT3 Expression	Conclusions	Ref.
**HYPERTENSION**									
Rat Wistar Male	pressure overload by abdominal aorta ligation	sham	LV	↑	↑	Tyr705	– (NC)	pressure overload increases STAT3 activation and phosphorylation	[65]
**CHRONIC KIDNEY DISEASE**									
Rat Sprague-Dawley Male	doxorubicin-induced CKD sedentary	normal kidney function sedentary	whole heart	N.D.	↑	?	N.D.	doxorubicin-induced CKD increases STAT3 phosphorylation	[71]
	doxorubicin-induced CKD swimming	doxorubicin-induced CKD sedentary	whole heart	N.D.	↓	?	N.D.	swimming decreases STAT3 phosphorylation in doxorubicin-induced CKD	
**SMOKING**									
Hamster N.A. Male	young (6-week-old) hamsters with secondhand cigarette smoke exposure (10 cigarettes for 30 min, 4 weeks)	young hamsters without secondhand cigarette smoke exposure	LV	N.D.	N.D.	N.A.	–	secondhand smoking does not alter STAT3 expression in young hamsters	[72]
	aged (72-week-old) hamsters with secondhand cigarette smoke exposure (10 cigarettes for 30 min, 4 weeks)	aged hamsters without secondhand cigarette smoke exposure	LV	N.D.	N.D.	N.A.	↑ (NC)	STAT3 expression showed a tendency of increase in aged hamsters due to secondhand smoking	
Rat Sprague-Dawley Male	young (6-week-old) rats with secondhand cigarette smoke exposure (10 cigarettes for 30 min, twice a day for 4 weeks)	young rats without secondhand cigarette smoke exposure	LV	N.D.	N.D.	N.A.	↑	secondhand smoking increases STAT3 expression in young rats	[73]
	aged (18-month-old) rats with secondhand cigarette smoke exposure (10 cigarettes for 30 min, twice a day for 4 weeks)	aged rats with without secondhand cigarette smoke exposure	LV	N.D.	N.D.	N.A.	↑ (NC)	STAT3 expression showed a tendency of increase in aged rats due to secondhand smoking	
**ALCOHOL**									
Mouse Wild-type friendly virus B Male	4% alcohol liquid diet for 12 weeks	regular liquid diet (without ethanol)	ventricles	↓	↓	Ser727	–	chronic 4% alcohol liquid diet decreases STAT3 activation and phosphorylation	[74]
Mouse Transgenic overexpressing *ALDH2* Male	4% alcohol liquid diet for 12 weeks	regular liquid diet (without ethanol)	ventricles	–	–	Ser727	–	chronic 4% alcohol liquid diet does not alter STAT3 activation, phosphorylation and expression in mice overexpressing ALDH2	
		wild-type 4% alcohol liquid diet for 12 weeks	ventricles	↑	↑ (NC)	Ser727	–	chronic 4% alcohol liquid diet increases STAT3 activation and does not alter STAT3 expression	

↓ in green cells: decrease; ↑ in red cells: increase; – in blue cells: no change; NC: not confirmed; N.D.: not detected; N.A.: not applicable; ?: phosphorylation site was not specified; LV: left ventricle; STZ: streptozotocin; p-STAT3: phosphorylated STAT3; t-STAT3: total STAT3; CKD: chronic kidney disease; ALDH2: aldehyde dehydrogenase 2.

**Table 5:** Effect of aging on cardiac STAT3 under non-ischaemic baseline conditions.

Animal or Cell	Test Group	Control Group	Tissue Sample	p-STAT3/t-STAT3 Activation	p-STAT3 Phosphorylation		t-STAT3 Expression	Conclusions	Ref.
Mouse *C57Bl6/J* Female	aged >13 months	young (<3 months)	RV	– (NC)	↓	Ser727	↓	age decreases STAT3 phosphorylation and expression	[18]
Mouse *C57Bl6/J* Female/Male	aged 21 months	young (8 weeks)	LV *mitochondrial fraction*	N.D.	N.D.	N.A.	↓	age decreases STAT3 expression	[80]
Rat Sprague-Dawley Male	aged 24 months	young (6 months)	LV	↓	↓ (NC)	Tyr705	– (NC)	age decreases STAT3 activation and reduced STAT3 activation may contribute to age-associated hypertrophy	[81]
	adult 12 months			↓	↓ (NC)	Tyr705	– (NC)		
Mouse *C57BL/6J* Male	aged 14 months	young (2 months)	whole heart	–	–	Tyr705	↑	age does not alter STAT3 activation and phosphorylation, but increases STAT3 expression	[82]
Rat Sprague-Dawley Male	aged 20‒24 months	young (3‒4 months)	whole heart	–	– (NC)	Ser727	– (NC)	age does not influence STAT3 activation	[83]
Mouse *C57BL/6J* Male	aged 24 months	young (3 months)	whole heart	– (NC)	–	Tyr705	–	age does not influence STAT3 phosphorylation and expression	[84]
Hamster N.A. Male	aged 72 weeks	young (6 weeks)	LV	N.D.	N.D.	N.A.	↑	age increases STAT3 expression	[72]
Rat Sprague-Dawley Male	aged 18 months	young (6 weeks)	LV	N.D.	N.D.	N.A.	↑	age increases STAT3 expression	[73]

↓ in green cells: decrease; ↑ in red cells: increase; – in blue cells: no change; NC: not confirmed; N.D.: not detected; N.A.: not applicable; RV: right ventricle; LV: left ventricle; p-STAT3: phosphorylated STAT3; t-STAT3: total STAT3.

**Table 6:** Effect of diabetes on cardiac STAT3 in settings of ischaemia/reperfusion.

Animal or Cell	Test Group	Control Group	Tissue Sample	p-STAT3/t-STAT3 Activation	p-STAT3 Phosphorylation		t-STAT3 Expression	Conclusions	Ref.
Rat Sprague-Dawley Male	in vivo regional (LAD) 30 min/120 min I/R in STZ-induced diabetes	I/R nondiabetic	ischaemic zone	↓ (NC)	↓	Tyr705	–	diabetes decreases post-ischaemic STAT3 phosphorylation	[93]
			LV	↓	↓	Tyr705 Ser727	– (NC)	diabetes decreases post-ischaemic STAT3 activation and phosphorylation	[29]
			whole heart	↓	↓	Tyr705	– (NC)	diabetes decreases post-ischaemic STAT3 activation and phosphorylation	[31]
			ventricles	↓	↓	Tyr705 Ser727	–	diabetes decreases post-ischaemic STAT3 activation and phosphorylation	[92]
Rat Sprague-Dawley Male	in vivo regional (LAD) 30 min/90 min I/R in STZ-induced diabetes	I/R nondiabetic	ischaemic zone	↓	↓	Tyr705	– (NC)	diabetes decreases post-ischaemic STAT3 activation and phosphorylation	[94]
Rat Sprague-Dawley Male	ex vivo global 30 min/120 min I/R in STZ-induced diabetes	I/R nondiabetic	LV	↓	↓ (NC)	Tyr705	– (NC)	diabetes decreases post-ischaemic STAT3 activation	[33]
Mouse leptin receptor null, homozygous db/db Male	ex vivo global 30 min/60 min I/R in high-fat-diet-induced diabetes	I/R C57BL/6J wild-type mouse	whole heart	↓	↓	Tyr705	↓	diabetes decreases post-ischaemic STAT3 activation, phosphorylation and expression	[95]
Rat Sprague-Dawley Male	isolated adult diabetic rat cardiomyocytes subjected to SI/R	isolated adult nondiabetic rat cardiomyocytes subjected to SI/R	cells	↓	↓	Tyr705	– (NC)	diabetes decreases post-ischaemic STAT3 activation and phosphorylation	[33]
H9c2 cells	high glucose conditions + 6 h/12 h SI/R	normal glucose conditions + 6 h/12 h SI/R	cells	↓	↓	Tyr705 Ser727	– (NC)	high glucose condition decreases post-ischaemic STAT3 activation and phosphorylation	[29]
		high glucose conditions	cells	↓	↓	Tyr705	– (NC)	high glucose condition decreases post-ischaemic STAT3 activation and phosphorylation	[32]
Rat Sprague-Dawley Male	isolated adult rat cardiomyocytes subjected to high glucose conditions + 45 min/2 h SI/R	normal glucose conditions + 45 min/2 h SI/R	cells	↓	↓ (NC)	Tyr705	– (NC)	high glucose condition decreases post-ischaemic STAT3 activation	[92]
H9c2 cells	high glucose conditions + 45 min/2 h SI/R	normal glucose conditions + 45 min/2 h SI/R	cells	↓	↓ (NC)	Tyr705	– (NC)		

↓ in green cells: decrease; ↑ in red cells: increase; – in blue cells: no change; NC: not confirmed; LV: left ventricle; STZ: streptozotocin; LAD: left anterior descending coronary artery; I/R: ischaemia/reperfusion; SI/R: simulated ischaemia/reoxygenation; p-STAT3: phosphorylated STAT3; t-STAT3: total STAT3.

**Table 7:** Effect of obesity, chronic kidney disease, aging, gender and depression on cardiac STAT3 in settings of ischaemia/reperfusion.

Animal or Cell	Test Group	Control Group	Tissue Sample	p-STAT3/t-STAT3 Activation	p-STAT3 Phosphorylation		t-STAT3 Expression	Conclusions	Ref.
**OBESITY**									
Mouse leptin -receptor-deficient (ob/ob) obese Male	leptin-receptor-deficient (ob/ob) obese mice + heart failure induced by coronary artery ligation	non-obese + heart failure induced by coronary artery ligation	whole heart	↓ (NC)	↓	Tyr705	↓	ob/ob obesity decreases STAT3 phosphorylation and expression in heart failure	[58]
Rabbit New Zealand white Male	in vivo regional 30 min/10 min I/R in diet-induced hypercholesterolemic rabbits	IR in normo- cholesterolemia	whole heart	–	– (NC)	Tyr705	– (NC)	diet-induced hypercholesterolemia has no effect on post-ischaemic STAT3 activation	[98]
**CHRONIC KIDNEY DISEASE**									
Rat Wistar Male	in vivo regional (LAD) 25 min/120 min I/R in 5/6 nephrectomy-induced CKD	I/R sham	whole heart	–	–	Tyr705	–	I/R has no effect on STAT3 in 5/6 nephrectomy-induced CKD	[99]
**AGING**									
Mouse C57Bl6/J Female	in vivo regional 30 min/10 min I/R in aged mice	young I/R	LV	↓	↓ (NC)	Ser727	– (NC)	age decreases post-ischaemic STAT3 activation	[18]
Rat Sprague-Dawley Male	in vivo regional 30 min/15 min I/R in aged rats	young I/R	whole heart	–	– (NC)	Ser727	– (NC)	age does not influence post-ischaemic STAT3 activation	[83]
**GENDER**									
Mouse *C57BL/6* Female/Male	ex vivo global 20 min/60 min I/R in male mice	I/R in female mice	whole heart	↓	↓	Tyr705	– (NC)	post-ischaemic STAT3 activation and phosphorylation is lower in male mice	[106]
				N.D.	N.D.	N.A.	↓ *	post-ischaemic STAT3 mRNA expression is lower in male mice	
Rat Sprague-Dawley Female/Male	ex vivo global 25 min/40 min I/R in male mice	I/R in female mice	whole heart	↓	↓ (NC)	Tyr705	– (NC)	post-ischaemic STAT3 activation is lower in male rats	[107]
**DEPRESSION**									
Rat Sprague-Dawley Male	ex vivo regional (LCA) 35 min/10 min I/R in chronic mild stress (3-week-long)-induced depression	I/R non-depressed	LV	–	– (NC)	Tyr705	– (NC)	depression does not alter post-ischaemic STAT3 activation	[109]

↓ in green cells: decrease; ↑ in red cells: increase; – in blue cells: no change; NC: not confirmed; N.D.: not detected; N.A.: not applicable; LV: left ventricle; LAD: left anterior descending coronary artery; LCA: left coronary artery; I/R: ischaemia/reperfusion; SI/R: simulated ischaemia/reoxygenation; CKD: chronic kidney disease; *: mRNA expression; p-STAT3: phosphorylated STAT3; t-STAT3: total STAT3.

**Table 8:** Effect of diabetes on cardiac STAT3 activation due to cardioprotective strategies against ischaemia/reperfusion.

Animal or Cell	Test Group	Control Group	Tissue Sample	p-STAT3/t-STAT3 Activation	p-STAT3 Phosphorylation		t-STAT3 Expression	Conclusions	Ref.
Rat Sprague-Dawley Male	in vivo regional (LAD) 30 min/120 min I/R in STZ-induced diabetes + morphine	I/R nondiabetic + morphine	ischaemic zone	↓ (NC)	↓	Tyr705	–	diabetes attenuates morphine-induced post-ischaemic STAT3 phosphorylation, and morphine cannot enhance post-ischaemic STAT3 phosphorylation in diabetes, which may contribute to the abrogation of morphine-induced cardioprotection in diabetes	[93]
		I/R in STZ-induced diabetes	ischaemic zone	– (NC)	– (NC)	Tyr705	– (NC)		
	ex vivo global 30 min/120 min I/R in STZ-induced diabetes + isoflurane postconditioning (PostC)	I/R nondiabetic + isoflurane PostC	LV	↓	↓ (NC)	Tyr705	– (NC)	diabetes attenuates post-ischaemic STAT3 activation due to isoflurane PostC, and isoflurane PostC cannot enhance post-ischaemic STAT3 activation, which may contribute to the abrogation of cardioprotection by isoflurane PostC in diabetes	[33]
		I/R in STZ-induced diabetes	LV	–	– (NC)	Tyr705	– (NC)		
	in vivo 30 min/90 min I/R in STZ-induced diabetes + sevoflurane PostC	I/R nondiabetic + sevoflurane PostC	area at risk	↓ (NC)	↓ (NC)	Tyr705	– (NC)	the activation of STAT3 due to sevoflurane PostC is lower when applied in diabetes, and sevoflurane PostC cannot enhance post-ischaemic STAT3 activation both in diabetic rats	[94]
		in vivo 30 min/90 min I/R in STZ-induced diabetes	area at risk	–	– (NC)	Tyr705	– (NC)		
	in vivo 30 min/120 min I/R in STZ-induced diabetes + ischaemic PostC	I/R nondiabetic + ischaemic PostC	whole heart	↓ (NC)	↓ (NC)	Tyr705	– (NC)	diabetes abrogates post-ischaemic STAT3 activation due to ischaemic PostC	[31]
		I/R in STZ-induced diabetes	whole heart	–	– (NC)	Tyr705	– (NC)		
Rat Sprague-Dawley Male	in vivo 30 min/120 min I/R in STZ-induced diabetes + repeated non-invasive limb ischaemic preconditioning	I/R nondiabetic + repeated non-invasive limb ischaemic PostC	LV	↓ (NC)	↓ (NC)	Tyr705 Ser727	– (NC)	the activation of STAT3 is lower in repeated non-invasive limb ischaemic preconditioning when applied in diabetes, the STAT3 activation is increased due to preconditioning in diabetes	[29]
		I/R in STZ-induced diabetes	LV	↑	↑ (NC)	Tyr705 Ser727	– (NC)		
H9c2 cells	6 h/12 h SI/R under high glucose conditions + remote time-repeated hypoxic preconditioning	normal glucose condition + remote time-repeated hypoxic preconditioning	cells	↓ (NC)	↓ (NC)	Tyr705 Ser727	– (NC)	the activation of STAT3 is lower in remote time-repeated hypoxic preconditioning when applied in diabetes, the STAT3 activation is increased due to remote time-repeated hypoxic preconditioning in high glucose conditions	[29]
		high glucose conditions + 6 h/12 h SI/R	cells	↑	↑	Tyr705 Ser727	– (NC)		
Mouse leptin receptor null, homozygous db/db Male	ex vivo global 30 min/60 min I/R in db/db diabetes + rapamycin	I/R nondiabetic + rapamycin	whole heart	–	–	Tyr705	–	rapamycin increases (restores) STAT3 activation, phosphorylation and expression in diabetes	[95]
		ex vivo global I/R 30 min/60 min in db/db diabetes	whole heart	↑	↑	Tyr705	↑		

↓ in green cells: decrease; ↑ in red cells: increase; – in blue cells: no change; NC: not confirmed; LV: left ventricle; STZ: streptozotocin; LAD: left anterior descending coronary artery; I/R: ischaemia/reperfusion; SI/R: simulated ischaemia/reoxygenation, PostC: postconditioning; p-STAT3: phosphorylated STAT3; t-STAT3: total STAT3.

**Table 9:** Effect of cardioprotective agents against ischaemia/reperfusion on cardiac STAT3 in diabetes.

Animal or Cell	Test Group	Control Group	Tissue Sample	p-STAT3/t-STAT3 Activation	p-STAT3 Phosphorylation		t-STAT3 Expression	Conclusions	Ref.
Mouse leptin receptor null, homozygous db/db Male	ex vivo global 30 min/60 min I/R in type II diabetes + Rapatar	I/R in type II diabetes	whole heart	↑	↑	Tyr705	– (NC)	Rapatar treatment induces post-ischaemic STAT3 phosphorylation in diabetes	[114]
Rat Sprague-Dawley Male	in vivo 30 min/90 min I/R in STZ-induced diabetes + N-acetylcysteine	in vivo 30 min/90 min I/R in STZ-induced diabetes	area at risk	↑	↑ (NC)	Tyr705	– (NC)	N-acetylcysteine enhances post-ischaemic STAT3 activation in diabetes	[94]
	in vivo 30 min/90 min I/R in STZ-induced diabetes + sevoflurane postconditioning + N-acetylcysteine	in vivo 30 min/90 min I/R in STZ-induced diabetes	area at risk	↑	↑ (NC)	Tyr705	– (NC)	STAT3 activation induced by sevoflurane postconditioning + N-acetylcysteine is superior to sevoflurane postconditioning or N-acetylcysteine in diabetes	[94]
		in vivo 30 min/90 min I/R in STZ-induced diabetes + sevoflurane postconditioning		↑	↑ (NC)	Tyr705	– (NC)		
		in vivo 30 min/90 min I/R in STZ-induced diabetes + N-acetylcysteine		↑	↑ (NC)	Tyr705	– (NC)		
	in vivo regional (LAD) 30 min/120 min I/R in STZ-induced diabetes + N-acetylcysteine + allopurinol	I/R in STZ-induced diabetes	ventricles	↑	↑ (NC)	Tyr705 Ser727	– (NC)	N-acetylcysteine + allopurinol increases (preserves) post-ischaemic STAT3 activation in diabetes	[92]
	isolated adult rat cardiomyocytes subjected to 45 min/2 h SI/R under high glucose conditions + adiponectin	high glucose conditions + 45 min/2 h SI/R	cells	↑	↑ (NC)	Tyr705	– (NC)	adiponectin increases post-ischaemic STAT3 activation in high glucose conditions	
	isolated adult rat cardiomyocytes subjected to 45 min/2 h SI/R under high glucose conditions + N-acetylcysteine + allopurinol			↑	↑ (NC)	Tyr705	– (NC)	N-acetylcysteine + allopurinol increases (preserves) post-ischaemic STAT3 activation in high glucose conditions	
H9c2 cells	45 min/2 h SI/R under high glucose conditions + N-acetylcysteine + allopurinol	high glucose conditions + 45 min/2 h SI/R	cells	↑	↑ (NC)	Tyr705	– (NC)		
	12 h/6 h SI/R under high glucose conditions + propofol	high glucose conditions + 12 h/6 h SI/R		↑	↑	Tyr705	– (NC)	propofol enhances STAT3 activation in settings of SI/R under high glucose conditions	[32]

↓ in green cells: decrease; ↑ in red cells: increase; – in blue cells: no change; NC: not confirmed; LV: left ventricle; STZ: streptozotocin; LAD: left anterior descending coronary artery; I/R: ischaemia/reperfusion; SI/R: simulated ischaemia/reoxygenation; p-STAT3: phosphorylated STAT3; t-STAT3: total STAT3.

**Table 10:** Effect of cardioprotective strategies against ischaemia/reperfusion on cardiac STAT3 activation in obesity, chronic kidney disease, aging and depression.

Animal or Cell	Test Group	Control Group	Tissue Sample	p-STAT3/t-STAT3 Activation	p-STAT3 Phosphorylation		t-STAT3 Expression	Conclusions	Ref.
**OBESITY**									
Rabbit New Zealand White Male	in vivo regional 30 min/10 min I/R in diet-induced hypercholesterolemic rabbits + oleuropein	I/R in diet-induced hypercholesterolemic rabbits	whole heart	↑	↑ (NC)	Tyr705	– (NC)	oleuropein increases post-ischaemic STAT3 activation in diet-induced hypercholesterolemia	[98]
**CHRONIC KIDNEY DISEASE**									
Rat Wistar Male	5/6 nephrectomy-induced CKD + ischaemic preconditioning	I/R in 5/6 nephrectomy-induced CKD	whole heart	↑	↑	Tyr705	–	ischaemic preconditioning increases STAT3 activation and phosphorylation in 5/6 nephrectomy-induced CKD	[99]
**AGING**									
Mouse *C57Bl6/J* Female	in vivo regional I/R + ischaemic postconditioning (PostC) in aged mice	young I/R + ischaemic PostC	LV	↓	↓ (NC)	Ser727	– (NC)	STAT3 activation is lower in aged rats subjected to ischaemic PostC, which may contribute to the age-related loss of ischaemic PostC-induced protection	[18]
Rat Sprague-Dawley Male	in vivo regional I/R + sevoflurane-PostC in aged rats	young I/R + sevoflurane-PostC	whole heart	–	– (NC)	Ser727	– (NC)	age does not influence STAT3 activation in sevoflurane-PostC	[83]
**DEPRESSION**									
Rat Sprague-Dawley Male	ex vivo regional (LCA) 35 min/10 min I/R in depression induced by chronic mild stress (3-week) + ischaemic PostC	I/R non-depressed + ischaemic PostC	LV	↓	↓ (NC)	Tyr705	– (NC)	the activation of STAT3 due to ischaemic PostC is abrogated when applied in chronic mild stress	[109]
		I/R in chronic mild stress-induced depression	LV	–	– (NC)	Tyr705	– (NC)		

↓ in green cells: decrease; ↑ in red cells: increase; – in blue cells: no change; NC: not confirmed; LV: left ventricle; STZ: streptozotocin; LCA: left coronary artery; I/R: ischaemia/reperfusion; SI/R: simulated ischaemia/reoxygenation; CKD: chronic kidney disease; PostC: postconditioning; p-STAT3: phosphorylated STAT3; t-STAT3: total STAT3.
